# Prognosis and regulation of an adenylyl cyclase network in acute myeloid leukemia

**DOI:** 10.18632/aging.103357

**Published:** 2020-06-22

**Authors:** Si-Liang Chen, Fang Hu, Da-Wei Wang, Zhe-Yuan Qin, Yang Liang, Yu-Jun Dai

**Affiliations:** 1 Department of Hematologic Oncology, Sun Yat-Sen University Cancer Center, Guangzhou, China; 2 State Key Laboratory of Oncology in South China, Guangzhou, China; 3 Collaborative Innovation Center for Cancer Medicine, Guangzhou, China; 4 National Research Center for Translational Medicine, Ruijin Hospital Affiliated to Shanghai Jiao Tong University School of Medicine, Shanghai, China

**Keywords:** prognostic value, AML, ADCYs factors, MAPK signaling pathway, 3D genome

## Abstract

We explored the roles of adenylyl cyclases (ADCYs) in acute myeloid leukemia (AML). Expression ADCYs in AML and their effect on prognosis was analyzed using data from Oncomine, GEPIA and cBioPortal databases. Frequently altered neighbor genes (FANGs) of *ADCY*s were detected using the 3D Genome Browser, after which the functions of these FANGs were predicted using Metascape tools. Cell viability and apoptosis were assessed using CCK-8 and Annexin V-FITC/PI kits. Expression levels of *ADCY*s were higher in AML cells lines and in bone marrow-derived mononuclear cells from AML patients than in control cells, and were predictive of a poor prognosis. A total of 58 *ADCY* FANGs were identified from the topologically associating domains on the basis of the Hi-C data. Functional analysis of these FANGs revealed abnormal activation of the MAPK signaling pathway. Drug sensitivity tests showed that fasudil plus trametinib or sapanisertib had a synergistic effect suppressing AML cell viability and increasing apoptosis. These findings suggest that dysregulation of *ADCY* expression leads to altered signaling in the MAPK pathway in AML and that the *ADCY* expression profile may be predictive of prognosis in AML patients.

## INTRODUCTION

Acute myeloid leukemia (AML) is a heterogeneous hematological malignancy [1, 2]. Three types of gene mutations are thought to play major roles in the pathogenesis of classical AML. Types I and II mutations are related to cellular proliferation and differentiation, while type III mutations affect genes encoding epigenetic factors involved in the pathogenesis and progression of AML [3].

Adenylyl cyclases (ADCYs) have been attracting increased attention in recent years [4]. These enzymes, which catalyze the generation of cAMP from ATP [5, 6], differ in their responses to upstream regulatory pathways and their distribution, and play essential roles in learning, synaptic plasticity, cardiovascular responses and tumorigenesis [7–10]. The nine members of the ADCY family (ADCY1-ADCY9) exhibit distinct responses to G protein coupled receptors and have been grouped into three subgroups based on their functional activities and sequence homology. Group 1 consists of ADCY1, ADCY3 and ADCY8, which are mainly distributed in neuronal tissues and stimulated by Ca^+2^/calmodulin [10]. Group 2 contain ADCY2, ADCY4 and ADCY7, which are Ca^+2^- independent and are stimulated by G proteins [11]. Group 3 includes ADCY5 and ADCY6, which are mainly expressed in heart and brain and are suppressed by G proteins [12]. In addition, there is ADCY9, which exhibits limited expression and is distinct from the other isoforms in that it is not activated by forskolin [13].

Aberrant expression of these isoforms can lead to changes in receptor-mediated activation of ADCYs, as well as alterations in the downstream signaling pathways [14]. However, the clinical impact of abnormal ADCY expression and its prognostic value has rarely been explored [15]. Dysregulated expression of ADCYs has been identified in colorectal cancer (CRC), hepatocellular carcinoma (HCC), prostate cancer, pancreatic cancer and cervical cancer [11, 16–18]. However, the mechanism underlying the abnormal ADCY expression seen in hematopoietic malignancies and their functional analysis has not yet been fully elucidated.

In the present study, we used bioinformatics analysis with online public data to explore possible functions of ADCYs and their unique prognostic value in AML. We also examined the regulation of ADCY-related pathways as potential targets for therapeutic intervention.

## RESULTS

### Transcription of *ADCY*s in AML

**Figure 1:**
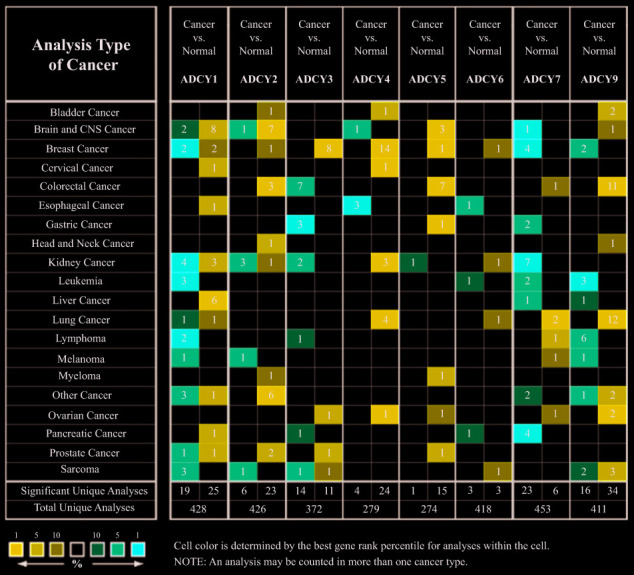
**Transcription of ADCYs within tumors (Oncomine).**

We initially used Oncomine databases to compare ADCY expression in various tumors and in controls (Figure 1). We found that *ADCY*s were highly expressed in cases of kidney tumor and leukemia. In Stegmaier’s dataset, a 3.002-fold increase in *ADCY1* expression was detected in AML as compared to control tissues. Similar increases in *ADCY1* expression in AML were also seen in Haferlach’s dataset (4.766-fold), Valk’a Leukemia Statistics (1.503-fold), and Andersson’s Leukemia Statistics (1.298-fold) (Table 1). Elevated *ADCY2* expression (1.034-fold) was also detected in AML patients vs. control samples. Consistent with that finding, Valk et al.’s data showed that *ADCY2* was upregulated 1.823-fold in AML, while Stegmaier et al. showed it to be upregulated 1.8331-fold. In addition, *ADCY3* expression was upregulated 1.312-fold in AML vs. control, and *ADCY5* and *ADCY6* expression was upregulated 1.025-fold and 1.105-fold, respectively. In Haferlach’s dataset, *ADCY9* expression increased 1.292-fold in AML, while Valk Leukemia Statistics showed a 1.137-fold increase (Table 1).

**Table 1:** The significant transcriptional changes of *ADCY*s in AML(Oncomine).

	**Fold Change**	***p* Value**	**t Test**	**Source and/or Reference**
*ADCY1*	3.002	0.001	3.917	Stegmaier Leukemia Statistics
	1.503	0.029	2.243	Valk Leukemia Statistics
	4.766	0.00000244	1.05	Haferlach Leukemia Statistics
	1.298	0.048	1.806	Andersson Leukemia Statistics
*ADCY2*	1.034	0.009	2.522	Haferlach Leukemia Statistics
	1.823	0.009	2.894	Valk Leukemia Statistics
	1.831	0.046	1.664	Stegmaier Leukemia Statistics
*ADCY3*	1.312	2.73E-27	12.339	Haferlach Leukemia Statistics
	1.223	0.036	2.085	Valk Leukemia Statistics
*ADCY4*	NA	NA	NA	NA
*ADCY5*	1.025	0.000799	3.24	Haferlach Leukemia Statistics
*ADCY6*	1.105	0.00018	3.678	Haferlach Leukemia Statistics
*ADCY7*	NA	NA	NA	NA
*ADCY9*	1.292	0.0000000000917	6.758	Haferlach Leukemia Statistics
	1.137	0.022	2.27	Valk Leukemia Statistics

NA, not available.

### Prognosis analysis of *ADCY*s in AML

**Figure 2:**
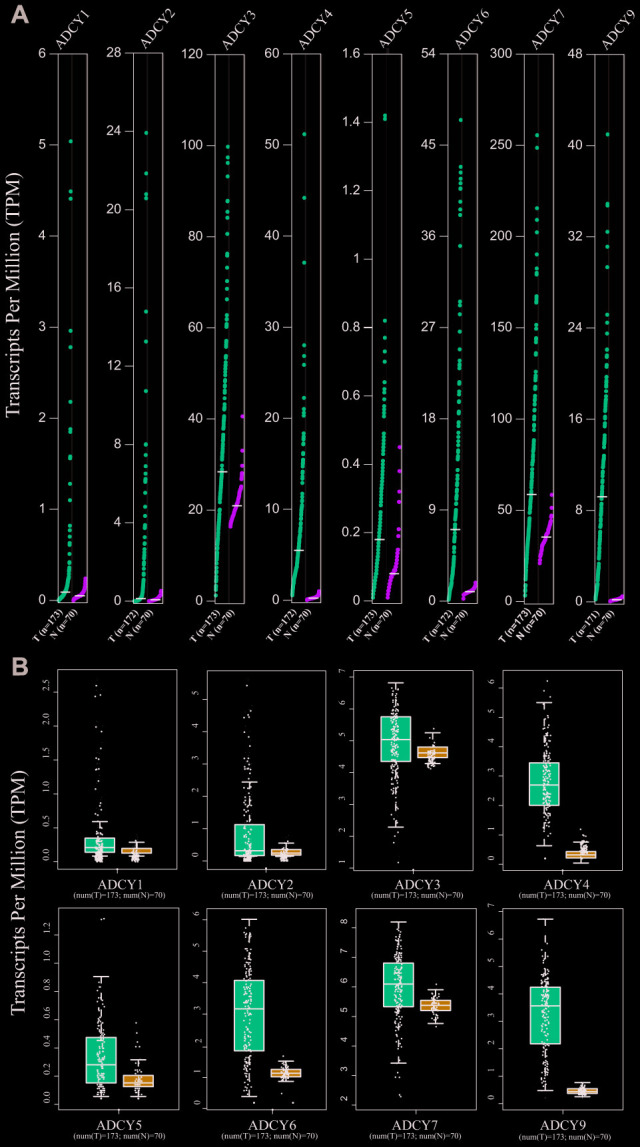
**Expression of ADCYs in AML and normal samples (GEPIA).** (**A**) The expression levels of ADCYs in AML compared with normal samples. (**B**) The TPM values of ADCYs in AML and normal samples. T represents AML samples and N represents normal samples.

**Figure 3:**
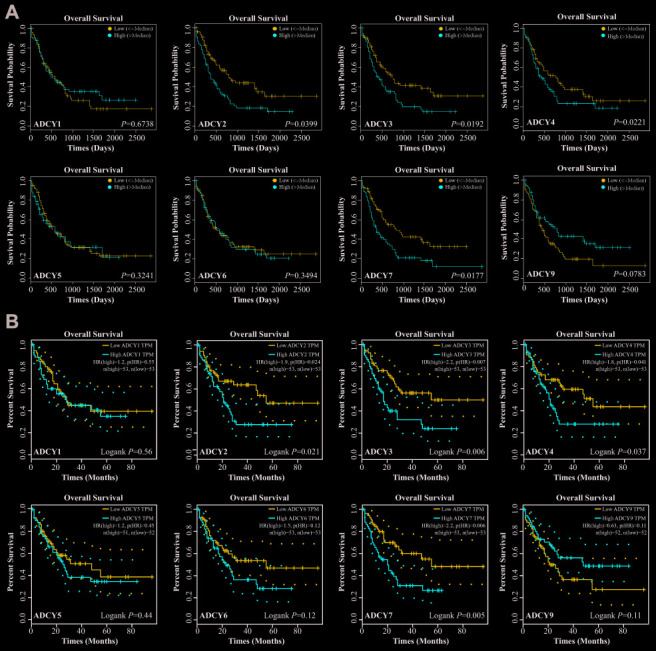
**Significance of ADCYs in predicting the prognosis for AML patients.** (**A**) The prognostic values of ADCYs in LinkedOmics datasets. (**B**) The prognostic values of ADCYs in GEPIA.

Expression levels of *ADCY*s between AML and matched control data from The Cancer Genome Atlas (TCGA) and the Genotype-Tissue Expression (GTEx) databases were compared on the basis of the Gene Expression Profiling Interactive Analysis (GEPIA) dataset (http://gepia.cancer-pku.cn/). These results showed that the expression of genes for all 9 ADCY isoforms was higher in AML samples than in control samples (Figure 2A, 2B). In addition, the association between *ADCY* expression and AML patient survival was explored using the LinkedOmics website (http://www.linkedomics.org/login.php). Using Kaplan-Meier analysis, we found that increased mRNA expression of *ADCY2* (P = 0.03994), *ADCY3* (P = 0.01924), *ADCY4* (P = 0.02211), and *ADCY7* (P = 0.01772) were significantly associated with poor overall survival (OS) in AML patients (Figure 3A). In addition, decreased levels of ADCY9 mRNA tended to indicate a poorer prognosis (P=0.078), though the effect was not statistically significant. Analysis of the GEPIA dataset revealed that median OS was shorter in AML patients showing higher expression of *ADCY2*, *3*, *4*, and *7* (Figure 3B).

We next used TCGA datasets to analyze the relationship between the common genetic and epigenetic mutations and ADCY expression. The samples were divided into two groups based on their mutations in AXSL1, CEBPA, FLT3, IDH1, IDH2, KIT, MLL, NPM1, RAS, TET2 and WT1 and then compared ADCY expression levels between the wild type (WT) and mutation (MUT) groups (Supplementary Table 2). We detected greater ADCY1 expression in the CEBPA WT group than the MUT group (p=0.005), whereas expression of ADCY9 was higher in the MUT group (p<0.001). For NPM1, expression levels of ADCY1 (p=0.02), ADCY2 (p<0.001) and ADCY4 (p=0.009) were much higher in MUT than WT group, while expression of ADCY3 (p<0.001) and ADCY6 (p=0.006) was higher in WT group. There were also scattered differences among these mutations. For example, expression of ADCY2 was higher in the FLT3 MUT group (p<0.001), and ADCY4 was more highly expressed in the IDH1 and IDH2 MUT groups (p=0.019 and p<0.001 respectively). Analysis of the relationships between the three subgroups of ADCY isoforms and these common mutations revealed no significant correlation with gene mutations (Supplementary Table 2). We therefore suggest that there is little correlation between these gene mutations in AML.

### Enrichment analysis of the frequently altered neighbor genes (FANGs) of *ADCY*s in AML

**Figure 4:**
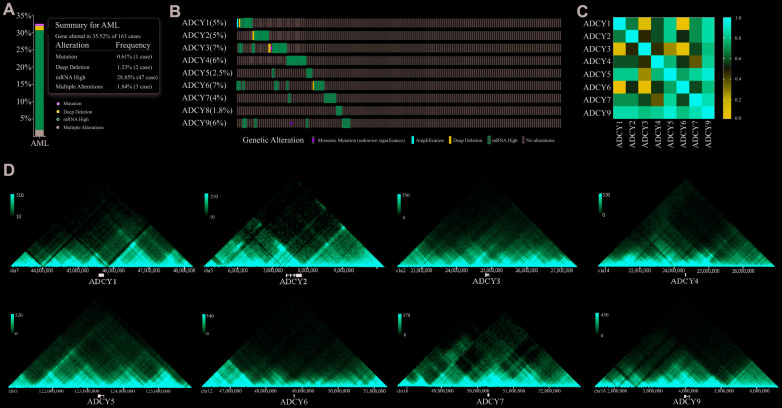
**Visual summary of ADCY alterations.** (**A**) Summary of ADCY alterations in AML (cBioPortal). (**B**) Details of ADCY alterations in AML (cBioPortal). (**C**) Gene relevance analysis among the ADCY family. (**D**) 3D genome of ADCYs in THP1 cells.

We investigated *ADCY* alterations, gene relevance, and the interaction networks using the cBioPortal database for AML (TCGA Provisional; http://www.linkedomics.org/admin.php). *ADCY* alterations were detected in 32.52% of 163 AML patients (Figure 4A, 4B). In addition, exploration of the relevance of *ADCY*s showed the potential for positive gene relevance among the *ADCY* family (Figure 4C).

**Figure 5:**
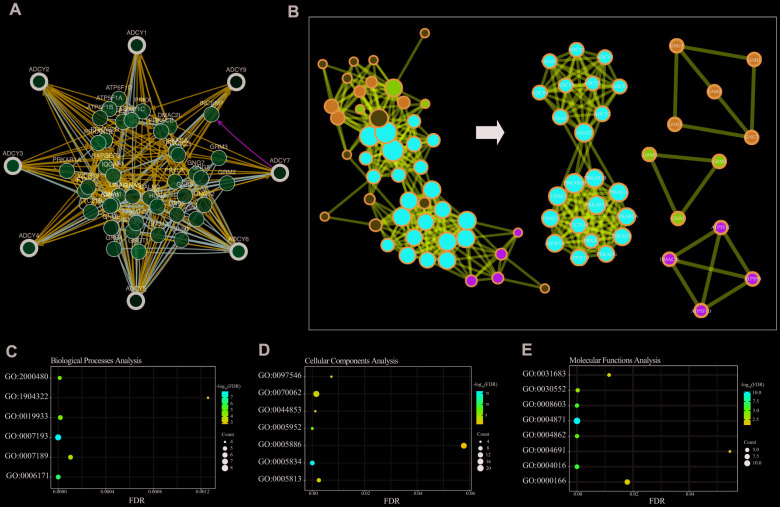
**Enrichment analysis of ADCY FANGs in AML patients.** (**A**) Network for ADCYs and the top 58 FANGs (cBioPortal). (**B**) Detailed net-structure of ADCY proteins in AML (Metascape). Bubble diagrams showing the top 58 FANGs in AML. (**C**) Biological processes. (**D**) Cellular components. (**E**) Molecular functions.

We then constructed a network for *ADCY*s using the 3D Genome Browser (http://3dgenome.org). This enabled us to simultaneously examine the gene regulatory events and the 3D genomic organization. Topologically associating domains (TADs) of *ADCY*s were identified through analysis of Hi-C data from THP1 cells, which enables us to predict the potential target genes (Figure 4D). The top 58 FANGs were screened in combination with the related *ADCY* genes reported in the cBioPortal dataset (Figure 5A). The results showed that genes involved in energy metabolism, including *ATP5F1A*, *ATP5F1B*, *CMPK1*, *GNAI1* and *GNAI2*, were closely associated with *ADCY*s (Supplementary Table 1). In addition, Figure 5B shows the protein-protein interactome network determined using Metascape (http://metascape.org/gp/index.html). The neighborhood protein network, where proteins were densely connected, was identified using the MCODE algorithm.

The Database for Annotation, Visualization and Integrated Discovery (DAVID) (https://david.ncifcrf.gov/summary.jsp) was used to predict potential functional pathways of *ADCY*s as well as their associated genes. Biological processes analysis showed that the cAMP-mediated signaling (GO: 0019933), the cAMP biosynthetic process (GO: 0006171), and the adenylate cyclase – activating/inhibiting G-protein coupled receptor signaling pathway (GO: 0007193 and 0007189) were significantly regulated by *ADCY*s in AML (Figure 5C). Using molecular functions analysis, we also found that cAMP-dependent protein kinase inhibitor/regulator activity (GO: 0008603, 0004862 and 0004691), cAMP binding (GO:0030552), and G-protein beta/gamma-subunit complex binding (GO: 0031683) were highly enriched in the *ADCY*s in AML (Figure 5E). In addition, cellular components analysis showed that plasma membrane/membrane raft (GO: 0005886 and 0044853), heterotrimeric G-protein complex (GO: 00058334), and cAMP-dependent protein kinase complex (GO: 0005952) were also significantly regulated by *ADCY*s (Figure 5D). Most of these were genes known to be associated with energy metabolism.

### Predicted therapeutic targets for *ADCY*s in AML

**Figure 6:**
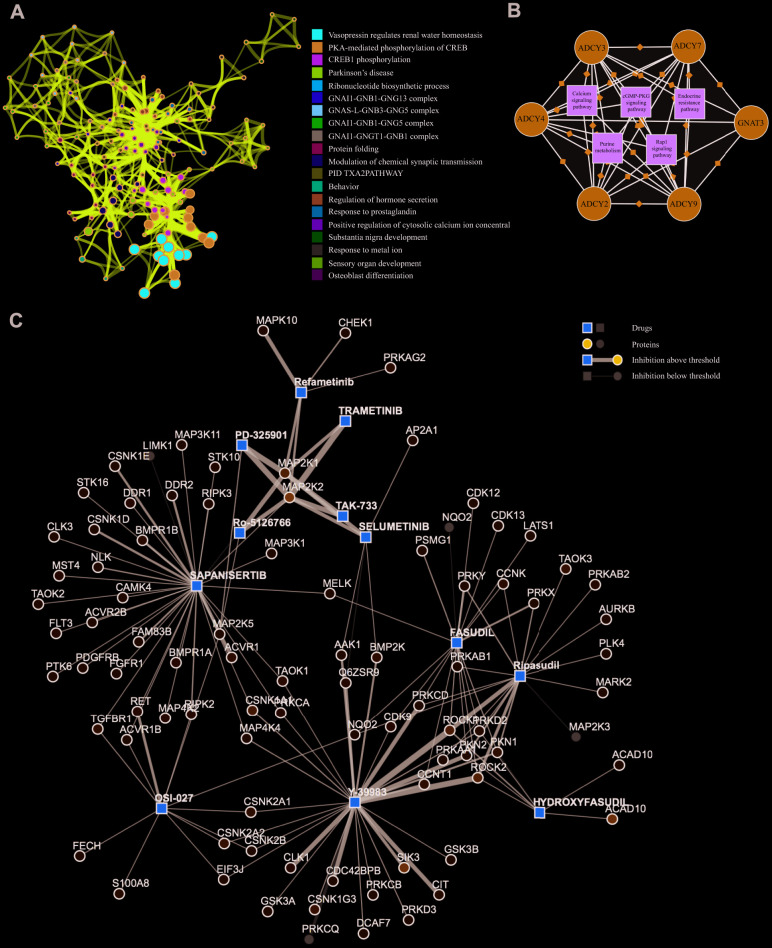
**KEGG Enrichment and therapeutic targets of ADCYs in AML.** (**A**) Significant terms among the gene memberships (Metascape). (**B**) KEGG analysis of FANGs in AML. (**C**) Protein-drug interaction map for inhibitors of the MAPK pathway in AML.

Significant terms among the gene membership profiles were identified using the Metascape tools and then hierarchically divided into a tree based on Kappa-statistical resemblance (Figure 6A). Next, Kyoto Encyclopedia of Genes and Genomes (KEGG) analyses were carried out to examine the pathways related to ADCY and FANG functions using the proteomicsdb dataset (https://www.proteomicsdb.org/). We found these genes were enriched in six important pathways related to AML leukemogenesis: endocrine resistance pathway, purine metabolism pathway, the Calcium signal transduction pathway, the MAPK signaling pathway, the cGMP-PKG signal transduction pathway, and the Rap1 signal transduction pathway (Figure 6B). Among those, the MAPK signaling pathway was found to be critical. The protein-drug interaction map suggested that MAPK signaling pathway inhibitors, including ROCK inhibitors (Fasudil, Y-39983 and Ripasudil), MEK inhibitors (Trametinib, TAK-733, Selumetinib, Ro-5126766, Refametinib, Pimasertib and PD-325901), and mTOR inhibitors (Sapanisertib, OSI-027) were associated with a tightly connected network that could potentially mediate therapeutic effects in AML (Figure 6C).

### Sensitivity effect and synergistic effect of inhibitors in AML

**Figure 7:**
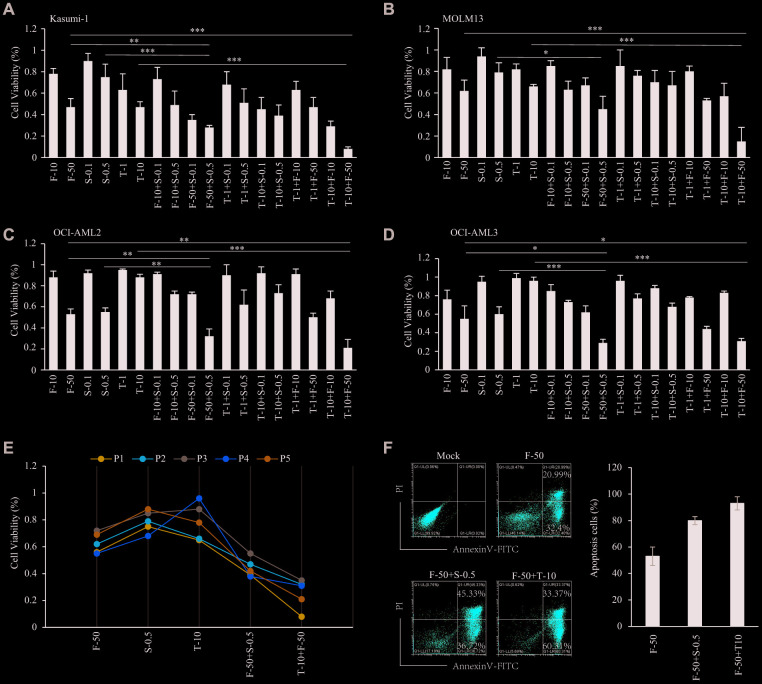
**Sensitivity effect of inhibitors in AML.** (**A**–**D**) Sensitivity effect of fasudil, trametinib and sapanisertib in AML cell lines of Kasumi-1 (**A**), MOLM13 (**B**), OCI-AML2 (**C**) and OCI-AML3 (**D**). (**E**) Sensitivity effect of inhibitors in AML patient samples. (**F**) Apoptosis induced by inhibitors among AML patient cells.

From among the abovementioned drugs, we chose three inhibitors that are currently in clinical trials and assessed their effects on the viability of cells from four AML cell lines. Using fasudil (ROCK inhibitor), trametinib (MEK inhibitor) and sapanisertib (mTOR inhibitor) with Kasumi-1, MOLM13, OCI-AML3 and OCI-AML2 cells, we found that all cells were sensitive to 50 μM fasudil, with about 50-60% of cells remaining viable after treatment for 48 h. Kasumi-1 cells were the most sensitive to trametinib (10 nM), with 50% of cells viable after 48 h (Figure 7A), and MOLM13 cells also showed sensitivity, with 60-70% remaining viable after 48 h (Figure 7B). By contrast, these drugs elicited no reduction in OCI-AML3 and OCI AML2 cell viability. However, both OCI-AML2 and OCI-AML3 cells were sensitive to 500 nM sapanisertib, with only 50-60% of cells remaining viable after 48 h (Figure 7C, 7D), which was consistent with previous reports [19]. Kasumi-1 and MOLM13 cells were less sensitive to 500 nM sapanisertib, with about 80% of cells remaining viable after 48 h. The IC50 of inhibitors in these AML cell lines were displayed in Table 2. Notably, AML cell viabilities could be significantly decreased by combining fasudil with trametinib or sapanisertib. The synergistic effect of fasudil plus trametinib was somewhat greater than fasudil plus sapanisertib. On the other hand, no synergistic effect on AML cell viability was seen with trametinib plus sapanisertib (Table 2).

**Table 2:** IC50 and combination index values in AML cell lines.

**Treatment**	**Kasumi-1**	**MOLM13**	**OCI-AML2**	**OCI-AML3**
Fasudil (IC50)	37.8μM	53.1μM	47.9μM	52.2μM
Sapanisertib (IC50)	2.1μM	1.3μM	0.4μM	0.6μM
Trametinib (IC50)	3.8nM	8.5nM	19.7nM	14.2nM
F-50μM + S-0.5μM (Combination index)	0.65	0.81	0.71	0.68
T-10nM + S-0.5μM (Combination index)	0.86	0.93	1.06	1.03
T-10nM + F-50μM (Combination index)	0.48	0.34	0.27	0.22

Given the ability of fasudil, trametinib and sapanisertib to reduce the viability in AML cell lines, we next sought to validate the effects of these inhibitors using mononuclear cells isolated from the bone marrow of primary AML patients. Consistent with the results from cell lines, patient cells treated with fasudil and trametinib or sapanisertib were less viable than cells treated with a single inhibitor. The synergistic efficiency of fasudil plus trametinib was greater than that of fasudil plus sapanisertib (Figure 7E). Consistent with those results, annexin V and PI staining showed that single inhibitors induced apoptosis among AML patient cells and that this effect was enhanced by treatment with fasudil plus trametinib or sapanisertib (Figure 7F).

## DISCUSSION

Dysregulation of ADCY expression has been reported in various solid cancers [11, 13, 15, 17], but the role of ADCYs in the development and progression of those cancers remains unclear, and bioinformatics analysis in AML is still lacking [20]. To explore the potential functions of ADCYs and their regulatory network in AML, we used the data published online to gain further insight into AML. Examination of both the Oncomine and TCGA datasets revealed that levels of ADCY expression are higher in AML patients than in control subjects. Additionally, we found that ADCYs may have prognostic values in AML, and that among the ADCY, high expression of group 1 (ADCY3, not ADCY1) and group 2 (ADCY2, ADCY4 and ADCY7) was associated with a poorer prognosis for all AML samples. Among the ADCY gene family members, ADCY1 is the most extensively investigated in solid cancers, where it is highly expressed in cancer tissues and related to a poor prognosis [10]. By contrast, ADCY1 expression did not affect the prognosis of AML patients, suggesting the actions of group 2 adenylyl cyclases (ADCY2, ADCY4 and ADCY7) in AML differ from those in other cancers.

Functional analysis suggested that cAMP may affect extracellular signaling in several tumors [12]. In the present study, the 3D genome organization of the THP1 leukemic cell line was explored using Hi-C technology. A total of 58 FANGs that were the most frequently altered in hematopoietic malignances were selected for further cluster analysis. Our results suggest that the MAPK signaling pathway is central among the six pathways involved in the leukemogenesis of AML. Furthermore, construction of a protein-drug interaction map revealed possible therapeutic strategies for treatment of AML using ROCK, MEK and mTOR inhibition to alter signaling in the MAPK pathway.

Smoking is thought to be a major risk factor for AML in older adults and childhood leukemia [21, 22]. We therefore sought to investigate the relationship between smoking and ADCY expression in AML [23]. However, information about smoking status is not available in either TCGA or the beatAML database [24]. We did find a case-control study that mentions the relationship between clonal hemopoiesis, therapy related myeloid malignancies, and smoking status [25]; unfortunately, those investigators did not perform RNA-seq or gene array analyses with these patients. Nonetheless, our findings are insufficient to shed light on the relationship between the ADCY expression profile and prognosis in patients with AML.

## CONCLUSIONS

Our findings suggest dysregulation of ADCY expression leads to altered signaling in the MAPK pathway in AML, and that the ADCY expression profile may be predictive of prognosis in AML patients.

## MATERIALS AND METHODS

### Patient samples

Between 2019 and 2020, a total of 5 AML patients newly diagnosed at the Sun Yat-sen University Cancer Center were enrolled in this study. All participants provided written informed consent in accordance with the regulations of the Institutional Review Boards of the Hospitals in agreement with the Declaration of Helsinki.

### Reagents

Inhibitors fasudil (S1573), trametinib (S2673) and sapanisertib (S2811) were obtained from Selleck Chemicals LLC (Houston, TX).

### Cell viability and apoptosis assay

AML cell lines (Kasumi-1, OCI-AML3, OCI-AML2 and MOLM13) were obtained from Da-Wei Wang (Ruijin hospital) and Da-Jun Yang’s lab (Sun Yat-sen University Cancer Center) and cultured in RPMI-1640 (Gibco, NY) supplemented with 10% fetal bovine serum (Biochrom AG, Berlin, Germany). Cells were seeded into 96-well plates at a density of 10^5^ cells/well. To determine the cytotoxicity of the inhibitors tested, cells were separately incubated with appropriate concentrations of each inhibitor. After 48 h, 10 μL of reagent from a Cell Counting Kit-8 (CCK-8, Dojindo Laboratories, Kumamoto, Japan) were added to each well. The samples were then incubated for an additional 4 to 6 h, at 37°C and the absorbance at 450 nm was measured using a spectrophotometer. An Annexin V FITC/PI staining kit (FA111-02, Transgen Biotech) was used to detect apoptotic cells induced by the inhibitors.

### Oncomine analysis

The Oncomine website (https://www.oncomine.org/re-source/login.html) was utilized to obtain ADCY gene expression data from cancer and control samples. ADCY expression was compared between clinical cancer specimens and paired normal controls using Student’s t test.

### GEPIA dataset

Thousands of normal and tumor specimens collected from the GTEx and TCGA were enrolled in the GEPIA dataset. Functional analyses, such as survival analysis and gene correlation analysis, were also carried out using this dataset.

### The c-BioPortal analysis

The cBioPortal (http://cbioportal.org) is an open-access website that contains over 225 tumor genomics datasets. ADCY alterations in the LAML samples were analyzed using this website. Genetic information, including mutations, gene splicing and copy number variations (CNVs), was also found in this dataset. The neighboring genes were excluded unless the frequencies were > 20%.

### Metascape analysis and the LinkeOmics dataset

Metascape provides a comprehensive gene function analysis. In this dataset, the MCODE algorithm was applied to identify the interacting network components and densely-related complexes, while Cytoscape is used to generate visualizations of these networks. LinkedOmics is also a publicly available portal that includes TCGA Cancer types. The relevance of differentially expressed genes among ADCYs was investigated in this dataset.

### 3D genome browser analysis

The Hi-C data from THP1 cells were analyzed using the 3D Genome Browser. In addition, TADs were identified to screen for potentially interacting genes in THP1 cells.

### The proteomicsDB dataset

The potential drug network was analyzed using the ProteomicsDB dataset. Selected from this website were MAPK pathway inhibitors, which exhibited the networks between the FANGs of ADCYs factors and the potential MAPK signaling pathway inhibitors.

### Combination index analysis

CompuSyn software (version 1.0; ComboSyn, Inc.Paramus, NJ, USA) were used to calculate the combination indexes according to the average fraction of viable cells in the cytotoxicity assays [26, 27]. The Combinatorial effects were classified into 4 parts: strong synergism for CI= 0.1–0.3; distinct synergism for CI = 0.3–0.7, mild synergism for CI = 0.7–0.9; additive for CI = 0.9–1.1.

## Supplementary Material

Supplementary Table 1

Supplementary Table 2
